# Hepatitis C Virus Protein Interaction Network Analysis Based on Hepatocellular Carcinoma

**DOI:** 10.1371/journal.pone.0153882

**Published:** 2016-04-26

**Authors:** Yuewen Han, Jun Niu, Dong Wang, Yuanyuan Li

**Affiliations:** 1 Xi’an Center for Disease Control and Prevention, Xi’an, China; 2 The General Hospital of Shenyang Military, Shenyang, China; 3 Air Force Aviation Medicine Identification and Training Center, Dalian, China; SAINT LOUIS UNIVERSITY, UNITED STATES

## Abstract

Epidemiological studies have validated the association between hepatitis C virus (HCV) infection and hepatocellular carcinoma (HCC). An increasing number of studies show that protein-protein interactions (PPIs) between HCV proteins and host proteins play a vital role in infection and mediate HCC progression. In this work, we collected all published interaction between HCV and human proteins, which include 455 unique human proteins participating in 524 HCV-human interactions. Then, we construct the HCV-human and HCV-HCC protein interaction networks, which display the biological knowledge regarding the mechanism of HCV pathogenesis, particularly with respect to pathogenesis of HCC. Through in-depth analysis of the HCV-HCC interaction network, we found that interactors are enriched in the JAK/STAT, p53, MAPK, TNF, Wnt, and cell cycle pathways. Using a random walk with restart algorithm, we predicted the importance of each protein in the HCV-HCC network and found that AKT1 may play a key role in the HCC progression. Moreover, we found that NS5A promotes HCC cells proliferation and metastasis by activating AKT/GSK3β/β-catenin pathway. This work provides a basis for a detailed map tracking new cellular interactions of HCV and identifying potential targets for HCV-related hepatocellular carcinoma treatment.

## Introduction

Hepatitis C virus (HCV) is a positive-strand RNA virus that causes of hepatitis C in humans [1]. More than 150 million people globally have chronic hepatitis C infection, and 350,000~500,000 people die each year from hepatitis C-related liver diseases [2]. Chronically infected patients present liver injury including hepatic steatosis, fibrogenesis, and insulin resistance, because of immune mechanisms and metabolic disorders. Long-term patients with HCV have a high risk of developing cirrhosis and hepatocellular carcinoma (HCC), but the molecular mechanisms of HCV pathogenesis are poorly understood [3].

The HCV genome contains 9.6 kb and encodes a polyprotein that is post-translationally processed into structural (CORE, E1, E2, and p7) and non-structural (F, NS2, NS3, NS4A, NS4B, NS5A, and NS5B) proteins [3]. These proteins binding of host proteins to form a complexes, which play an important role in HCV infection and hepatocarcinogenesis. Therefore, to understand the mechanisms of HCV pathogenesis and the relationship between HCC and HCV, it is important to determine the interactions between HCV proteins and human proteins. For example, the HCV NS2 protein and its interaction protein were investigated in the HCV life cycle [4]. NS4B interaction networks with host proteins were considered to understand the role of NS4B in the HCV life cycle and illuminate potential therapeutic targets [5]. Furthermore, the interactions of CORE and NS4B with human proteins have been analyzed toward understanding their role in HCC pathogenesis [6]. However, HCV pathogenesis are complex biological processes, and individual protein-protein interaction research is insufficient to elucidate the mechanism.

The rapidly growing PPIs data between viral and host have led to increased efforts in understanding the role of viral pathogenesis [7]. In particular, the availability of large-scale interaction data between viral and host proteins is beginning to be used to creat network-based models [8–11]. A network analysis approach to a virus-human PPI network revealed the highly connected of host interactors in cellular, which can clarify the new signaling pathway and the importance of “hub protein” in viral pathogenesis. Therefore, systematic study of the HCV-human interaction network, particularly in relation to HCC progression might provide clues toward achieving a decrease in the incidence of HCC and establishing effective treatments.

In this study, we attempted to catalogue all published interactions between HCV and human proteins to construct the HCV-human and HCV-HCC protein interaction networks. Based on systematic analysis method, we display the biological knowledge regarding the mechanism of HCV pathogenesis, particularly with respect to pathogens in HCC. Furthermore, we analyzed the importance of each protein in the HCV-HCC network using algorithm and predicted AKT1 as a critical protein in HCC progression. Moreover, we found that NS5A promotes HCC cell proliferation and metastasis by activating AKT/GSK3β/β-catenin pathway. We expect that this approach and analysis will help identify potential targets for future effective anti-HCV therapies.

## Material and Methods

### Text mining of human proteins that interact with HCV

Information about PPIs between HCV proteins and human proteins was collected from the VirusMint database [12], and HCVpro databases [13]. In addition, a detailed literature search was carried out on PubMed to supplement binary interactions between HCV and human proteins. Literature indexed in PubMed was searched using keywords [e.g., (“HCV” [title] OR “hepatitis C virus” [title] AND (“1980/01/01” [Date—Publication]: “2015/10/12” [Date—Publication])], and 28,152 published journal abstracts were identified. Further text analysis revealed 471 HCV-interacting proteins from 640 reports of putative interactions between HCV and human proteins. Finally, 455 unique interactors participating in 524 HCV-human interactions were identified for 11 HCV proteins (S1 Table).

### The integrated human interactome network

Human-human protein interaction data were collected from the BioGRID [14], MINT [15], and STRING databases [16]. NCBI official gene names were used to eliminate redundancy due to the existence of different protein name or alias. Finally, we collect 176 non-redundant PPIs in the human interactome (S2 Table).

### Random walk with restart (RWR) on the HCV-HCC network

RWR can be used to rank nodes in a graph [17]. It simulates a random walker who starts at a group of seed nodes and moves to a randomly chosen neighbor or jumps back to the seed nodes with a certain probability. After some number of steps, the walker reaches each node in the graph with a steady probability by which all nodes in the graph are ranked. The probability vector **p**_t_ of finding the random walker at each node at step *s*+1 is determined from

$${\text{P}}_{\text{t}+1}=\left(1-\gamma \right)M^{T}p_{\text{t}}+\gamma p_{0},$$

Where **p**_0_ represents the initial probability vector, *M* represents the transition matrix of the graph, and *γ* represents the restart probability. In this study, **p**_0_ = (1/n,1/n,…,1/n), where n is the number of proteins involved in PPIs, and *γ* was empirically determined as 0.6.

### Bioinformatics analysis and network visualization

The list of genes was submitted to DAVID online tools for gene ontology (GO) enrichment analysis (http://david.abcc.ncifcrf.gov/home.jsp). Functionally or structurally related proteins were clustered in the overrepresented biological annotation with statistic “Enrichment Score”. Cellular pathway data were retrieved from KEGG (http://www.kegg.jp/). The enrichment of a specific KEGG pathway was tested using Benjamini test to control the false discovery rate. HCV-human and human-human PPIs relationships were mapped and visualized in a network structure using Cytoscape (version 2.8.3) software [18].

### Cell culture and transfection

We obtained Huh7 cells from ATCC and grew them in DMEM medium supplemented with 10% fetal bovine serum and penicillin (100 U/mL) and streptomycin (100 μg/mL). To construction NS5A overexpressing cell line, the NS5A sequence was recombined into the destination vector pCDH-EF1-MCS-T2A-Puro. The pCDH-NS5A plasmids or pCDH plasmid (for negative control cells) was co-transfected with psPAX2 and pMD2.G packaging plasmids into 293FT cells to produce pseudo-viral particles. The resulting both pseudo virus were then used to infect Huh7 cells, respectively, and positive cells were screening by puromycin.

### Western blotting

Cellular protein lysates were boiled for 5 min and then the denatured samples (20μg) were separated in 4%–12% gradient NuPAGE^®^ NovexBis-Tris gels (Invitrogen). The separated proteins were transferred to PVDF membranes (Millipore) and probed with primary antibodies: NS5A (1:1500; Abcam), AKT (1:1000; CST), p-AKT (Ser473) (1:1000; CST), GSK3β (1:1000; CST), p-GSK3β (1:1000; CST), β-catenin (1:5000; Abcam) and β-actin ((1:1000; CST). The immune reactive bands were detected by staining with HRP-conjugated secondary antibodies (1:1000; CST).

### Cell-proliferation assays

To perform cell-proliferation assays, we seeded 2×10^3^ Huh7 cells (Blank), negative control cells (NC) or NS5A overexpression cells (NS5A) in 96-well plates and then quantified the proliferation by using the Cell Counting Kit-8 (CCK-8) (Beyotime) at 0, 12, 24 and 36 h. The CCK-8 solution was added to each well at various time points and incubated at 37°C for 2 h, after which the optical density was measured at 450 nm.

### Scratch-healing assay

Cells in complete media were plated in poly-D-lysine-coated 24-well plates (2 × 10^4^ cells/well). After overnight incubation, one artificial wound was scratched into the mono layers by using the tip of a 10-μL micropipette; after wounding, the cells were washed at least twice in PBS to eliminate floating cells. We monitored wound closure and photographed the cultures at 36 h post-wounding.

### Transwell-migration assay

We examined the invasive behavior of cells by performing transwell-migration assays. Briefly, 8-μm-pore-size Transwell inserts (Corning) were coated with 1.0 mg/mL Matrigel (BD) in cold serum-free medium. Cells (1 × 10^5^) were plated on the top side of the transwell insert (upper chamber), and serum-free medium was added to the bottom chamber. After 24 h, stationary cells were removed from the top side of the membrane, and the cells that had migrated to the bottom side of the inserts were stained with 0.1% crystal violet and counted under a light microscope. In each well, we counted cells in 5 fields and then calculated the mean number of cells per field. Each experiment was performed in triplicate and repeated at least twice.

### Statistical analysis

All data are presented as the mean ± SEM. GraphPad Prism 5 Software was used for statistical analysis. The two-tailed unpaired *t*-test was used for statistical analysis to evaluate the differences between 2 groups of samples, and *P* < 0.05 was considered statistically significant.

## Results and Discussion

### Text mining of HCV interaction protein

**Fig 1:**
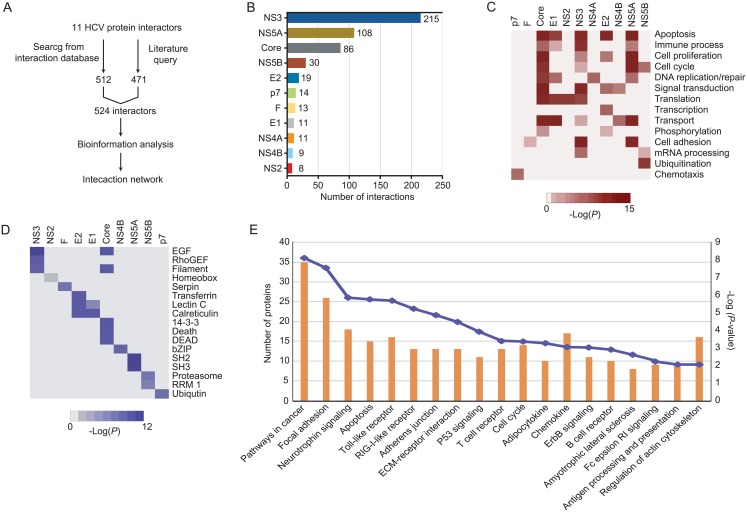
Systematic analysis of the interaction of HCV proteins. (A) Schematic illustration of the major steps in the HCV-human protein interaction network. (B) Distribution of interactions in the HCV-human bipartite interaction network with respect to the 11 HCV proteins. (C) Heat maps representing enriched biological functions of human proteins that interact with HCV proteins. (D) Heat maps representing enriched protein domains from human proteins that interact with HCV proteins. (E) Correlations between the enriched KEGG pathways (blue line) and the corresponding number of interactors (orange bars) for the HCV proteins.

Data on HCV-human PPIs were collected from PubMed and two protein interaction databases (VirusMint and HCVpro), which identified 471 and 512 HCV-targeted human proteins, respectively. After removing redundant interactions, 455 unique interactors participating in 524 HCV-human interactions were identified for 11 HCV proteins (Fig 1A, S1 Table). The distribution of interactions with respect to each of the HCV proteins were very different (Fig 1B). For example, HCV protein NS3 interacts with the most human proteins (215), whereas NS2 interacts with the fewest (8). Of the other HCV proteins, NS5A and CORE have reasonable numbers of interactions with human proteins (108 and 86, respectively). We used these 524 interactions among 11 HCV proteins for bioinformatics analysis and to map the PPI network between viral and host proteins.

### Systematic analysis of HCV-human complexes

The functional diversity of the identified host proteins is illustrated by hierarchical clustering based on GO biological process (GO-BP) classifications (Fig 1C). As the main function HCV proteins is virus infection, it is not surprising that many interactors have functions in cell adhesion, apoptosis, and immune processes. Furthermore, a large number of interactors are involved in cell proliferation, cell cycle, DNA replication/repair, and signal transduction, suggesting that interactors may be played an important role in carcinogenesis processes beyond viral invasion. An unexpectedly large number of interactors were involved in transport, phosphorylation, mRNA processing, ubiquitylation, and chemotaxis; these data suggested that HCV proteins may also play an important role in molecular processing.

We next clustered the occurrence of specific structural domains in the protein interactors (Fig 1D). Surprisingly, although HCV proteins are closely related, they interact with vastly different groups of host proteins. The EGF and filament domains were associated with NS3 and CORE, suggesting that they affect multiple pathways in liver inflammation and HCC [19, 20]. Remarkably, E1 and E2 interact with proteins that contain C-type lectin and calreticulin domains, suggesting that E1 and E2 have a previously unidentified preference for interacting with human proteins that is reminiscent of virus attachment to the host cell surface for infection [21], and correctly folded of HCV proteins [22].

To better understand the biological functions of HCV interactors, we determined the enrichment of specific pathways for all interactors. Of the 455 interactors, 425 (~93.4%) were mapped to 19 pathways (*P*< 0.01) using the KEGG human pathway database (Fig 1E). Interacting proteins that were highly enriched in cancer-related pathways (35 proteins, *P*< 10^−8^) may be closely related to HCC.

### Construction of an HCV-human interactome network

**Fig 2:**
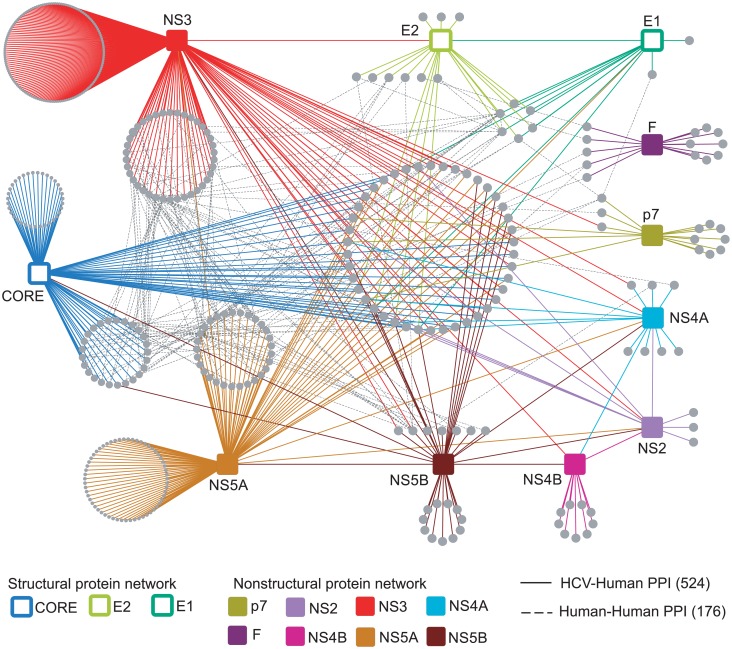
HCV and human protein interaction network. In total, 525 HCV-human interactions between 11 HIV proteins and 455 human proteins are shown. Colored squares: HCV proteins. Gray circles: human proteins. Solid edges: interactions between HCV proteins and human proteins. Dotted edges: interactions between human proteins. PPI: protein-protein interaction.

We next constructed a network representing the 524 HCV-human interactions with nodes corresponding to 11 HCV (squares) and 455 human factors derived from the databases (circles) (Fig 2). We also introduced 176 interactions between human proteins (dotted edges) derived from databases (S2 Table), which can help to identify many host complexes that have been previously characterized.

About forty of the identified human proteins interact with at least two HCV proteins (Fig 2, middle) and were classified as components of the core complex. Proteins within this core complex are clearly associated with virus infection, cirrhosis, and HCC. For example, two contained lectin-related domains (CLEC4M and CD209), which are shared by E1 and E2 proteins and are involved in viral entry in hepatocytes through induction of the p38-MAPK pathway [23]. VAPA and VAPB interact with NS5A or NS5B, which are thought to serve as a scaffold for the replication complex within lipid microdomains, and affect HCV infection and replication [24, 25]. Interestingly, SETD2 and HOXD8 interacted with 5 out of 11 HCV proteins, and these interactions may affect the histone methylation and transcriptional activation of regulation to promote the process HCC [26]. In addition to direct interaction between HCV proteins and human proteins, we observed the indirect interaction between them through sharing the third-part partners. For example, NS3 protein is not directly bound FAS in HCV-human protein network, but it can bind CASP8 to increase sensitivity of Fas-induced apoptosis [27].

### Functional analysis of the relationship between HCV and HCC

**Fig 3:**
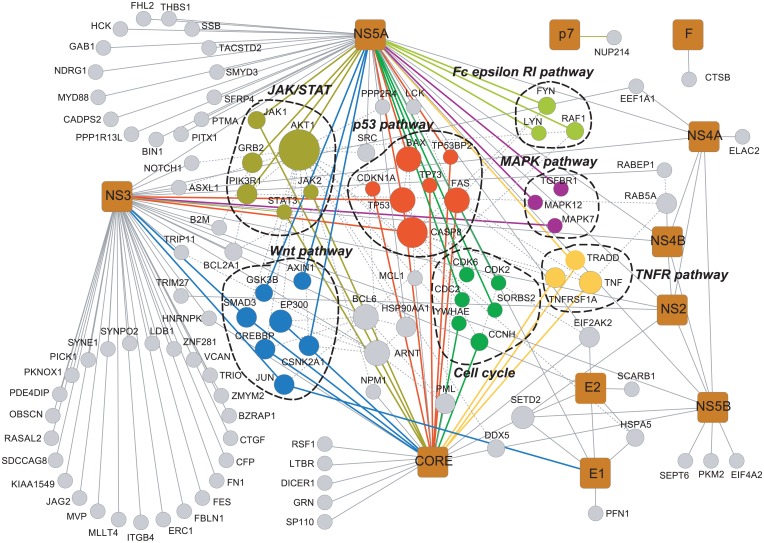
Network analysis of the relationship between HCC and HCV. The HCV-HCC interaction network contains nodes corresponding to 11 HCV and 110 unique human interactors constituting 128 HCV-HCC interactions. Squares: HCV proteins. Circles: human proteins. Circle sizes indicate the number of interacting proteins. Pathway-associated proteins are shown in various colors, and other proteins are shown in gray. Solid edges: interactions between HCV proteins and human proteins. Gray dashed edges: interactions between human proteins. Colored lines indicate different pathways.

Human hepatocarcinogenesis is closely related to HCV-induced dysregulation of the balance between survival and apoptosis [28]. To review the current understanding of how HCV dysregulates this balance in HCC, we extracted 110cancer-related proteins (S3 Table) from the data by matching the list of interactors with 3600 cancer genes from the COSMIC database [29]. We constructed the HCV-HCC interaction network containing nodes corresponding to 11 HCV and 110 unique human interactors forming 128 HCV-protein interactions (Fig 3). These interactors constitute several signaling pathways by KEGG pathway analysis, including the JAK/STAT, Wnt, TNF, MAPK, Fc epsilon RI, cell cycle, and p53 pathways, and mediate many cellular functions in HCC process (Fig 3).

The p53 pathway has been shown to mediate cellular stress responses in HCC; TP53 (p53) protein can initiate DNA repair, cell-cycle arrest, senescence and apoptosis [30]. In our HCV-HCC network, the CORE, NS3 and NS5A protein interact with p53 [31–33]; therefore, they may play a role in coordinating the balance between proliferation and programmed cell death through the p53 pathway. Studies have shown that CORE or NS3 binding to p53 inhibits cyclin-dependent kinase (CDK) inhibitor p21/Waf, which regulates the activities of cyclin/CDK complexes involved in cell cycle control and tumor formation [34]. NS5A forms a heteromeric complex with TATA box-binding protein (TBP) and p53, and the complex interferes with the binding of p53-TBP to target DNA sequences, which play an important role in transcriptional transactivation [35].

We also identified the TNF pathway as a novel pathway affected by HCV proteins in HCC progression. TNF pathway deregulation can perturb cellular proliferation, survival, differentiation, or apoptosis [36]. Some studies have demonstrated that CORE protein binding to TNFR1 and TRADD forms a ternary complex and activates TNF-induced JNK in a CORE concentration-dependent manner [37]. Furthermore, in human HepG2 cells, CORE or E2 can inhibit TNF-α-induced apoptosis by inhibiting activation of CASP8 [34]. Regardless of the mechanism, the aberrant gene expression and deregulation in TNF pathway ultimately generates a unique response by accelerating cell cycle, repressing apoptosis, and contributing to cell survival and oncogenesis.

### Assessment of the protein importance in the HCV-HCC protein network

The interaction network indicates connections between nodes (proteins); however, the importance of each node in the network is not equivalent. Therefore, understanding the importance of each node will increase understanding of the mechanism of HCC. To assess the importance of each protein in the network, we used an RWR algorithm to infer the HCV-HCC relationship [17]. It simulates a random walker who starts at a group of seed nodes and moves to a randomly chosen neighbor or jumps back to the seed nodes with a certain probability. After some number of steps, the walker reaches each node in the graph with a steady probability by which all nodes in the graph are ranked. By prioritizing candidate genes, we attempted to make better use of the phenotypic data.

Our predictions indicate the importance of each node in the network (S4 Table). The 10 most important proteins (Table 1) are mostly related apoptosis (CASP8, FAS, BAX, and TP53) and transcriptional regulation (ARNT, BCL6, SETD2, and TP53), which play a central role in hepatocarcinogenesis [34]. In addition, two proteins (RAB5A and NUP214) involved in the transport in HCC, suggest that they mediated TNFR1 internalization [38], and nuclear-cytoplasmic transport of proteins in HCC process [39]. Notably, AKT1, an important protein kinase involved in the phosphoinositide-3-kinase (PI-3K)/protein kinase B (AKT) pathway, received the highest score and may play a key role in the HCV-HCC network.

**Table 1:** Top ten important proteins in the HCV-HCC network.

Gene name	HCV proteins	RWR Score	Effect
AKT1	NS5A	0.245	Binding and activation of kinases
ARNT	NS3	0.110	Transcriptional regulation
CASP8	NS3	0.110	Increased sensitivity to Fas-induced apoptosis
BCL6	NS3	0.107	Transcriptional regulation
FAS	CORE	0.107	Apoptosis
RAB5A	NS4B	0.105	Trafficking
BAX	NS5A	0.103	Apoptosis inhibition
SETD2	E1, E2, CORE, NS2, NS5B	0.101	Transcriptional regulation
TP53	CORE, NS3, NS5	0.093	Apoptosis inhibition; transcriptional regulation
CTSB	F	0.083	Intracellular degradation; invasion and metastasis
NUP214	p7	0.083	Nuclear-cytoplasmic transporter activity

### NS5A promotes HCC cell proliferation and metastasis by activating AKT/GSK3β/β-catenin pathway

AKT1 is a crucial regulator of a number of cellular processes including proliferation, differentiation, and metastasis in several human cancers [40]. Several studies have shown that NS5A binds and activates PI-3K/AKT signaling and can inhibit apoptosis by disrupting p53-mediated apoptosis, inactivating CASP9 and BAD, and suppressing death receptor-mediated apoptosis in HCC [40]. In our HCV-HCC networks, we find that the interaction between NS5A and AKT may affect other downstream effectors, for example, GSK3β. GSK3β is a ser-thr kinase that is phosphorylated by the kinase AKT, and plays a critical role in determining the levels of β-catenin, which plays a pivotal role in tumor cell proliferation and metastasis [41].

**Fig 4:**
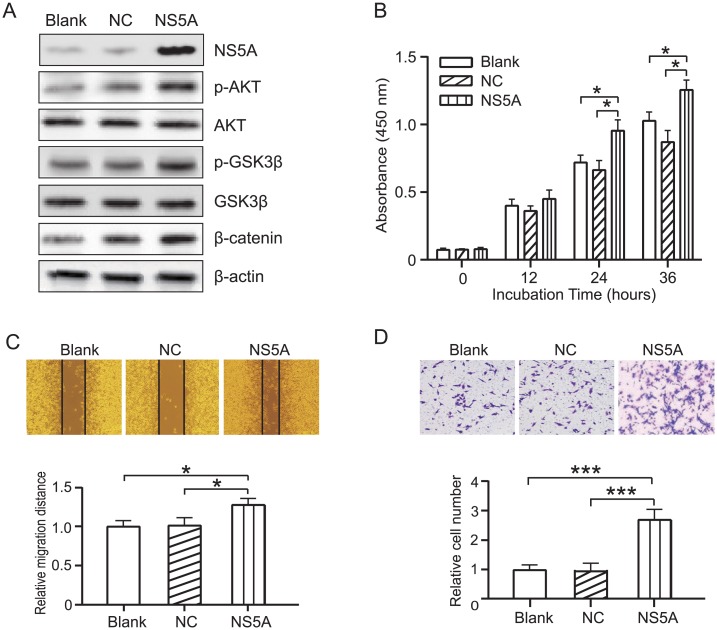
NS5A promotes HCC cell proliferation and metastasis by activating AKT/GSK3β/β-catenin pathway. (A) The p-AKT, AKT, p-GSK3β, GSK3β and β-catenin protein expression levels were determined by western blot analysis from Huh7 cells (Blank), negative control cells (NC) or NS5A overexpression cells (NS5A). β-actin was used as internal control for normalization in western blotting. (B) The proliferation of NS5A overexpression cells were evaluated using the CCK-8 assay (at 0, 12, 24, and 36 h). (C) The migration of NS5A overexpression cells by using a scratch-healing assay. (D) The invasiveness of NS5A overexpression cells compared with the control cells by using a Matrigel-transwell assay system. Five fields of cells were counted for each well, and the mean number of cells per field was calculated. All data shown are from 2 independent experiments conducted in triplicate. *P<0.05; *** *P*< 0.001

We explored whether NS5A promoted HCC cells proliferation and metastasis by mediating the AKT/GSK3β/β-catenin pathway. By western blot analysis, we found that NS5A overexpression in Huh7 cells elevated protein levels of phosphorylated AKT and GSK3β, while increasing downstream proliferation/metastasis-related target proteins β-catenin as compared to the control (Fig 4A). Next, we found that NS5A overexpression in Huh7 cells grew significantly more faster than control cells by using the CCK-8 assay analysis (Fig 4B). Next, to assess whether NS5A affects the migration/invasion of HCC cells, we examined the of Huh7 cells by using scratch-healing assay and transwell-migration assays. Results indicated significant enhanced cell migration/invasion effects was higher than that of control cells (Fig 4C and 4D). Collectively, these results suggest that NS5A might promote HCC cell proliferation and metastasis via modulating the AKT/GSK3β/β-catenin pathway.

## Conclusions

HCC is the fifth most common type of cancer, the third major cause of cancer-related death and a major cause of death in patients with chronic HCV infection, and is responsible for approximately one million deaths each year [2]. Because the role of HCV infection and the pathogenic mechanisms of the cancer-causing variant are not entirely clear, effective treatment for HCC remains a challenge.

In a complex biological system, we cannot study the biological function without correctly understanding their component parts and interactions. Therefore, systematic study interacting relationships between HCV proteins and human proteins can help us to clarify the mechanism of HCV infection and mediate HCC progression. In this study, through an in-depth analysis of the literature and databases, we identified 524 HCV interactors and mapped the global HCV-human PPI network, particularly in relation to HCC progression PPI network. Through screening and mapping HCV-HCC interactions, we found that about half of the interactors are involved in multiple signaling pathways such as the JAK/STAT, Wnt, TNF, MAPK, Fc epsilon RI, cell cycle, and p53 pathways, which are crucial in HCC oncogenesis. Moreover, we predicted the importance of each protein using an RWR algorithm and found that AKT1 may play a key role in the HCV-HCC network.

AKT is a serine protein kinase involved in several carcinogenetic mechanisms, e.g. cell survival, protein synthesis, apoptosis, genetic instability and cell cycle [40]. AKT could phosphorylate and inactivate GSK3β/β-catenin complex, but its participation in the Wnt signalling pathway is still controversial [41]. In this work, we found that NS5A promotes HCC cells proliferation and metastasis, probably by activating AKT/GS3KB/β-catenin pathway. This could lead to development of a new treatment strategy involving disruption of the interaction between NS5A and AKT/GSK3β/β-catenin pathway.

In conclusion, this work provides a basis for a detailed map tracking new cellular interactions of HCV and identifying potential targets for HCV-related hepatocellular carcinoma treatment.

## Supporting Information

S1 Table All interactors of HCV proteins. (XLSX)

S2 Table Human-human interactors in HCV-human network. (XLSX)

S3 Table HCC-related interactions of HCV proteins. (XLSX)

S4 Table The importance of interactors in HCV-HCC network. (XLSX)
